# Collective conceptualization of parental support of dual career athletes: The EMPATIA framework

**DOI:** 10.1371/journal.pone.0257719

**Published:** 2021-09-22

**Authors:** Kinga Varga, Ciaran MacDonncha, Laurence Blondel, Enrico Bozzano, Fabrice Burlot, Rute Costa, Nadine Debois, Dominique Delon, Antonio Figueiredo, Joerg Foerster, Masar Gjaka, Carlos Gonçalves, Flavia Guidotti, Caterina Pesce, Andrej Pišl, Eoin Rheinisch, Ana Rolo, Sara Rozman, Gary Ryan, Anne Templet, Antonio Tessitore, Giles Warrington, Laura Capranica, Mojca Doupona

**Affiliations:** 1 University of Ljubljana, Ljubljana, Slovenia; 2 Institute of Economics, Centre for Economic and Regional Studies (KRTK), Budapest, Hungary; 3 Department of Physical Education and Sport Sciences, University of Limerick, Limerick, Ireland; 4 Health Research Institute, University of Limerick, Limerick, Ireland; 5 INSEP, Paris, France; 6 CONI, Rome, Italy; 7 Ginàsio Clube Figueirense, Figueira da Foz, Portugal; 8 Faculty of Sport Science and Physical Education, University, University of Coimbra, Coimbra, Portugal; 9 University of Hamburg, Hamburg, Germany; 10 European Athlete as Student, Ghaxaq, Malta; 11 Department of Movement, Human and Health Sciences, University of Rome Foro Italico, Rome, Italy; 12 Department of Sport and Movement Science, University for Business and Technology, Pristina, Kosovo; 13 EUSA Institute, Ljubljana, Slovenia; 14 Sport Ireland Institute, Dublin, Ireland; Qatar University College of Education, QATAR

## Abstract

**Background:**

This study aimed to use a concept mapping methodology to develop a European framework of the needs of parents/guardians (P/G) for supporting athletes combining sport and education (dual career, DC).

**Methods:**

By means of a concept mapping methodology, 337 French, Irish, Italian, Portuguese, and Slovenian parents sorted and rated 80 potential statements associated to parenting DC athletes.

**Results:**

Five distinct clusters emerged: 1. P/G’ roles, needs and awareness to support athletes, including 22 statements (mean:3.7; range: 3.2–4.2 pt); 2. Requirements for effective planning of DC pathway, including 19 statements (mean:3.7; range: 3.2–4.5 pt); 3. Educational opportunity, including 13 statements (mean:3.5; range: 3.1–4.0 pt); 4. Policy and provision for DC, including 19 statements (mean:3.7; range: 3.1–4.2 pt); and 5. Athletes’ lifestyle & self-management, including 7 statements (mean:4.0; range: 3.5–4.5 pt). Estimates of effect size (Partial eta-squared) were calculated for ANOVAs to assess the degree of variability on the statement importance ranking as the dependent variable accounted for by the demographic data.

The concept mapping showed good validity (stress value: 0.11) and high reliability (rSHT: 0.99, rSHM: 0.98; rRR:0.98). One-third of the statements indicated differences (p<0.05) in relation to the P/Gs’ gender *and* the athletes’ education level, competition level and sport typology.

**Conclusion:**

In synthesizing the opinions, experience and needs of P/Gs of DC athletes the present framework provided sound theoretical underpinnings to inform the development of an online educational programme for empowering parenting DC athletes (https://edu.empatiasport.eu/eng/), as well as be a foundation for future Pan-European DC research on how these statements interact with each other, in different European contexts.

## Introduction

The European Union devotes significant and necessary attention to sport as a social and economic phenomenon contributing to its strategic objectives [1–3]. Consequently, the European Commission’s Directoreate-Generale of Innovation, Research, Culture, Education and Youth, and its Education, Audiovisual and Culture Executive Agency (EACEA) address European sport-related issues through several programmes aimed to develop evidence-based policy in the field of sport, to foster cooperation between sports stakeholders, to promote initiatives in support of active lifestyles, and to tackle the European socio-economic policy agenda in sport [4].

Since the European Year of Education through Sport 2004 (EYES 2004) sport has been recognized as an educational and social inclusive tool. In particular, the European White Paper on Sports [5] introduced the term “dual career” (DC) to highlight the athletes’ right to combine sport and education for pursuing their holistic development, which is grounded on two fundamental human rights: The right of education [6], and the right to play [7]. Actually, the combination of sport and education is a long-lasting process starting at young ages, spanning across the developmental years, and lasting into adulthood. Throughout these years, the typology, volume, intensity and organization of sport-specific and academic-specific demands vary significantly, being also subjected to the different cultural dimensions, welfare systems and DC opportunities of the Member States of the European Union [8–12]. In addition, effectively managing a DC will increase the potential for the athlete to successful engage with society and the labour market at the end of their competitive years and to reduce the risk of dropout from either an academic or sporting career due to conflicting demands [11,13]. In light of the different DC approaches and policies in place, the European Commission published the European Guidelines on Dual Careers of Athletes [14] and tackles its implementation in the Member States, also financing collaborative partnerships between DC stakeholders, which have different roles, responsibilities, and visions in supporting the educational and/or sport development of the athletes.

In recent years, a multidisciplinary scientific research focus is contributing significantly to the development of a European DC culture [15,16], and a revised position statement on the athlete’s career development and transitions has been prepared [13]. The holistic development the athlete as a student, a conceptual framework of the athlete’s DC transitions with regard to four main domains (e.g., athletic, psychological, psychosocial, and academic/vocational) has been proposed [17]. In addition, ecological models of the athlete’s DC represent the circumstances, relationships, environments, and their interactions at the micro (e.g., the individual athlete), the meso (e.g., parents, peers, teachers/employers, coaches, sport managers), the macro (e.g., sport clubs/federations, educational institutions, and labour market), and the policy (e.g., national and European governing bodies) level, which influence the student-athlete development [11,12].

In highlighting the supporters of their DC at personal, sport, and academic levels, elite student-athletes stressed the relevant role parents play, mainly acting at the meso and most proximal dimension of their support entourage [18] in fact, a positive parental role of DC athletes includes emotional, motivational, instrumental, material, social, and informational, and financial support [19]. According to personal experiences and opinions of parents regarding their support of student-athletes, complex demands of parenting DC athletes emerge) [20–25]. The thematic analysis of a recent systematic literature review on parenting DC athletes highlighted a two-level construct, encompassing individual and inter-individual aspects [26]. Within the individual level, the main themes pertained to the parents’ approach to both sport and education, and the parents’ coping capabilities to stressors, whereas the inter-individual level concerned the parents’ relationship with the athlete, and with the sport and academic environments. The authors concluded that there is a need to explore the interrelationships between relevant aspects affecting parenting DC athletes [26].

In considering the lack of a framework on parenting DC athletes [26,27] and the need of specific knowledge and skills necessary to facilitate positive relationships between parents and athletes and other stakeholders of the sport and educational entourages [26,28] the Erasmus+ Programme (Erasmus+ is the European Union’s programme to support education, training, youth and sport in Europe) supported a consortium of ten university and sport institutions with a consolidated relationship at national and European levels in DC from six Member States (Ireland, Italy, France, Malta, Portugal, and Slovenia) having an aim to structure an Education Model for Parents of Athletes in Academics (EMPATIA). In an effort to provide an effective parenting education and guidance for DC athletes, an on-line interactive multimedia programme was deemed appropriate to offer information specifically tailored to parents of student-athletes, and to reach a large target audience at relatively low cost. To inform the educational model, an evidence- and eminence-based approach was used to gain a comprehensive understanding of the key aspects, their structures and interrelationships, which are relevant for parents to effectively support DC athletes [29]. The development of effective educational programmes that appeal to European parents of DC athletes in relation to different sport-specific cultures and organizations, educational levels, and local, regional, and national DC policies, poses both a challenge and an opportunity. In particular, to construct a sound framework of parenting DC athletes, it is necessary that a diversity of experts, disciplines and relevant stakeholders provide inputs to ensure the identification and inclusion of the most salient theories, concepts and aspects [23,30].

Concept mapping is a methodology which effectively gathers, integrates and visually and numerically represents the composite thinking of a group of relevant and expert stakeholders around a complex social phenomenon, e.g., the needs of parents supporting DC athletes, human behaviours, intervention and policy implementation needs. The conclusions of a concept mapping exercise can be used to inform new theory development, to design new and effective interventions, policy and programmes, to inform evaluation methodologies and to increase our understanding of the nature, structure and interaction of aspects pertaining to societal processes. Concept mapping involves a methodological framework with a predefined sequence of research phases designed to organize and represent ideas based on the unique integration of qualitative and quantitative methods [30–33]. A range of methodologies (e.g. literature review, focus groups, expert consensus, etc.) can be used to generate and structure opening statements and to identify a list of potential aspects relevant to the question of interest. This step normally involves a small sample of stakeholders and a validation of the opening statements/aspects by experts. Subsequently the statements/aspects are sorted and rated by a more extensive sample of participants characterised by targeted end users. This step allows for the ranking and identification of distinct clusters of statements, which are represented in a two-dimensional concept map [33]. Clusters and statements located close to each other carry a similar meaning, whereas distant ones are less related. Statements with higher rating are deemed more relevant and important. Recently, this methodology has been applied to explore the complexity related to the development of on-line educational programmes [34,35] and to the frameworks of determinants of physical activity and sedentary behaviours [36,37] and sports practice [38–40].

Thus, the aim of the current study was to develop a conceptual framework of aspects relevant to the education and empowerment of the parents of European DC athletes to create a supportive environment for the athletes and to increase their confidence to successfully combine academic and sporting careers. The concept mapping methodology enables a synthesis of parents’ opinions on relevant aspects affecting parenting DC athletes. In particular, the framework will show how these aspects interact with each other. Thus, the framework will be applied to improve theory development and as a comprehensive and reliable knowledge base for the development of an educational programme for parents of DC athletes.

## Methods

The concept mapping methodology encompassed four key stages (Table 1): 1) preparation (terminology, protocol and inclusion criteria of experts); 2) generation of statements; 3) structuring (sorting and rating of statements) and 4) data analysis and interpretation. A range of protocols were used to inform and complete the concept mapping process: a systematic literature review, national focus groups, consensus and validation of DC experts, face to face interactions, and web-based surveys. In involving parents from five European countries having different DC policies (e.g., state-centric DC regulations: France and Portugal; laissez faire/no formal DC structures: Ireland, Italy and Slovenia) (Aquilina & Henry, 2010), the EMPATIA project intended to contribute to a European-wide understanding of the parent behaviours and the challenges of the parental role for an optimal support of DC athletes [29]. The Ethical Committee of the University of Ljubljana approved the EMPATIA project (9:2018). Participation in the study was voluntary and informed consent was assumed with subjects’ reply that they were willing to participate.

**Table 1:** Stages, content, time frame and participants of the concept mapping process.

Stage	Content	Time Frame	Participants
Preparation	Protocol and inclusion criteria of parents	Feb.—Apr. 2018	EMPATIA Team
Generation and validation of statements	Literature review	May—Sep. 2018	EMPATIA Team Parents
	Generation of statements (national focus groups)		EMPATIA Team DC experts
	Refinement of statements		
	Validation of statements		
Concept mapping	Sorting and rating of statements	Dec. 2018-Mar 2019	Parents
Data analysis and interpretation	Structure of the framework and final consensus	Apr.–Jun. 2019	EMPATIA Team

### Preparation

A protocol document and standard operating procedures (SOPs) was developed and agreed among the EMPATIA partners to ensure clear and consistent guidance and implementation for each stage of the concept mapping process. The protocol documents provided step by step details for each phase of the study and SOPs were defined for the following: parent focus group protocols and instructions for participants to complete the online concept mapping exercise. In particular, to develop national databases (France, Ireland, Italy, Portugal, Slovenia) of parents of DC athletes, inclusion criteria were agreed based on the athletes’ age (range: 14–24 yrs), sex (female and male), type of sports (individual or team), competitive level (national, international or both), and academic level (high school/secondary level or university/tertiary level). To facilitate the invitation of parents of DC athletes to participate in the concept mapping process, maximum targets of 200 parents for smaller nations (Ireland, Slovenia) and 300 for bigger nations (France, Italy, and Portugal) were agreed. The databases were built through personal networks, direct contact with national sports federations, coaches, and athletes, and held securely and in confidence.

A key aspect of the preparation stage was to identify and agree opening statements to underpin and direct the brainstorming during national focus group sessions with the parents. The following statements were informed by relevant extant literature [11,18,26,41] and were subsequently agreed and approved by the EMPATIA research team:

1. What kind of information on the athletes’ needs should be provided to parents to help them support the athlete’s dual career?
2. What kind of information on the sports environment should be provided to parents to help them support the athlete’s dual career?
3. What kind of information on the academic environment should be provided to parents to help them support the athlete’s dual career?
4. What kind of educational opportunities do you consider relevant to educate parents to support the athlete’s dual career?
5. What kind of information on dual career-related policies and services should be provided to parents to help them support the athlete’s combination of sports and academic careers (dual career)?

The questions were agreed to address the athlete’s needs, the parent’s challenges within the sport environment and the academic environment, the parent’s need of knowledge of DC policies and services, and the parent’s need of education opportunities.

### Generation and validation of statements

To generate a comprehensive, culturally relevant, evidence- and eminence-based list of potential statements to inform the EMPATIA framework, multiple methodologies were used. These involved: 1) the experiences, perceptions, opinions and needs of parents of DC athlete gathered via focus groups [23]; 2) a systematic literature review [26] and 3) the consensus opinion of the EMPATIA research team.

In total 12 focus groups were conducted across the five partner nations (France, Ireland, Italy, Portugal, and Slovenia). Informed consent was provided by 115 parents (49 mothers, 66 fathers) of athletes competing in 13 individual (i.e, athletics, canoeing, dancing, diving, equestrian, fencing, gymnastics, kayaking, modern pentathlon, sailing, shooting, synchronized swimming, and swimming) and 8 team (basketball, handball, hockey, rowing, rugby, soccer, volleyball, water polo) sports. The focus group sample was drawn from the established database of potential participants and was purposeful (i.e. based on the best balance across the database inclusion criteria e.g. athlete gender, sport, level of participation and academic level). The purpose of each focus group (2-hour duration) was to facilitate participants to brainstorm the five key opening questions with the desired outcome of collectively preparing and agreeing a set of statements describing the issues, problems, concerns and needs of dual career parenting education. The relevant statements identified by each of the 12 focus groups were collated under headings associated with each of the five opening questions: Athlete Needs; Sport Environment; Academic Environment; Education Opportunities and Policy & Services. The SOPs for the focus group methodology ensured representative opinion and consistency and rigour in implementation. Participation by parents from five different nations who support athletes in a range of different circumstances facilitated multiple opinions. An emphasis was placed on encouraging open discussion throughout the focus group sessions, listening to the participants and incorporating all their views without a judgemental fashion into the list of potential statements relevant for the EMPATIA education programme.

The agreed set of statements (i.e., short phrase or sentence) concluded by each focus group was registered on an excel sheet. To produce an equivalent translation of the original statement, two members of the EMPATIA research team from each country performed an independent translation into English and agreed a combined version, this version was then back translated by an English reviewer to confirm alignment with the original statements. All statements translated to English stemming from the focus groups were now placed on an excel sheet. A list of 59 statements drawn from a systematic literature review [26] were also included. All statements within each of the five headings were screened for repetitions, similarity and clarity in an unbiased fashion by an individual who was not an expert in DC. The conclusions of this refinement process were shared and confirmed by the leaders of the focus group methodology from each nation. A final and extensive list of 211 preliminary EMPATIA framework candidate statements was agreed: Athlete Needs (n = 68); Sport Environment (n = 49); Academic Environment (n = 37); Education Opportunities (n = 32) and Policy & Services (n = 25). This number of statements was deemed too many for an effective online concept mapping exercise and a further iteration of refinement was required. At a consensus meeting of the EMPATIA research team (N = 20) repetitions within and between each of the five main headings were eliminated; fragmented statements were condensed into related broader statements; the clarity and comprehension of all statements was improved and the relevancy of each statement to the aim of the EMPATIA project was confirmed. A list of 81 candidate statements for the EMPATIA framework was synthesized. A final validation exercise of these 81 statements was performed by 32 DC experts from 11 countries (i.e., Belgium, Brazil, Bulgaria, Croatia, Hungary, Italy, Latvia, The Netherlands, Portugal, Spain, and Sweden) who were participants at an international DC conference (European Athlete as Student, Coimbra (Portugal), September 2018) [23]. Participants were asked to rate the clarity of the statements by means of a 5-point Likert scale and also to suggest any additional statement which should be included. Four statements did not meet the clarity of threshold ≥ 4.0 pt. and were revised accordingly, two statements were collapsed into one and the comprehensive nature of the statements included was confirmed. A final list of 80 statements was generated.

### Structuring (sorting and rating)

The Ariadne online concept mapping platform was used (http://www.minds21.org/) to accommodate the two key concept mapping tasks of ranking the importance and sorting into clusters of all statements by the parents of DC athletes. Pilot trials of the data collection functionality and data transfer were run to ensure that all glitches were removed and that data collection was effectively and correctly occurring. To gain further insights, the participants were administered, an anonymous questionnaire. On their supporting role (mother/father/male or female guardian), time commitment (hr^.^week^-1^) to the sport and academic career of the athlete, and the financial support. In addition, information on the DC athlete’s nationality, sex, year of birth, education level (e.g., high school/secondary level or university/tertiary level), living arrangement (e.g., at home, away from home, or in athletic residence), sport discipline and competitive level (e.g., national or international or both) was obtained.

The participant sample for the concept mapping exercise was drawn from the established national databases of potential participants. A target of 600 potential participants (parents of DC athletes) was set across the five national partners. All potential participants were sent a pre-notification email which indicated the aim of the EMPATIA project, the intention to develop a framework for an educational resource to support parents of DC athletes, the time required and the nature of the concept mapping exercise, the potential that the task could be completed online at home or at a face to face meeting opportunity and an assurance that all responses will be anonymous and confidential. Subsequently a second communication was made with potential participants inviting them to complete the concept mapping exercise. This communication provided participants with detailed instructions and how to engage with the online concept mapping platform, a unique participant code, a link to the background questionnaire and a unique link to the concept mapping platform. Both background questionnaire data and the concept mapping responses were linked via the unique participant code. In the instances where parents of DC athletes engaged with the national sporting federations at face to face events, the opportunity was taken for parents to complete the relevant online data collections at this event. Participants were advised that their participation was entirely voluntary, that they could withdraw at any time without providing a reason, that their consent to participate was implied by submitting the online survey and that all responses were anonymous and confidential in nature.

The detailed instructions provided to all participants contained the following: a) a list of the 80 statements with a request to briefly read and become familiar; b) a notification of two key tasks which required completion. Task 1 Ranking of Statement Importance: Participants were asked to rank each statement according to the following question: “To what extent is the statement important for a dual career parenting education programme?” Ranking scale was 1–5 (1 = not important; 5 = very important). Each statement was depicted on a “card” in the online environment and participants were able to click and drag the statement card into a frame corresponding to the 1–5 importance ranking; Task 2 Clustering of Statements: Participants were asked search for similarities or patterns across the statements according to their own logic and reasoning to organise the statements into meaningful clusters. A maximum of 10 clusters was possible. Participants were asked to review and cross check their decisions before final submission of the statement ranking and clustering tasks. Furthermore, figures and images derived from the concept mapping platform provided specific and additional guidance.

In considering that online surveys including >20 items and demanding longer time commitment may result in a lower response rate [42], participants were advised to complete the tasks in two sessions separated by 1 day and two reminder emails were sent with a ten-day separation.

A pre-notification email informing on the aim of the EMPATIA project, the time required to complete the concept mapping exercise online or at a face-to-face meeting, assurance of anonymous and confidential results, and opportunity to withdraw at any time without providing a reason was sent to potential participants. Upon manifestation of interest, a second communication provided detailed instructions and a unique participant code to access the questionnaire and the concept mapping platform. In particular, the instructions encompassed: a) a request to read and become familiar with the 80 statements; b) to score on a 5-point scale (1 = not important; 5 = very important) “To what extent is the statement important for a DC parenting education programme?”; c) to organise the statements into meaningful clusters (a maximum of 10 clusters was allowed) according to their own logic and reasoning of similarities/patterns; d) to review and cross check their decisions before final submission. In considering that online surveys including >20 items and demanding longer time commitment may result in a lower response rate [42], participants were allowed to complete the tasks in separate sessions. Data collection extended from February, 26 to April 20, 2018.

### Data analysis

Prior to the computations, a concatenated dataset was obtained where the data of the background questionnaire and the importance ranking score for the statements (n = 80) from the concept mapping exercise were linked. Descriptive statistics for the demographic data as well as for the statement importance ranking were calculated (mean, standard deviation, and frequency of occurrence expressed in absolute values or percentages), and a chi-square test was applied to assess differences (p>0.05) between frequencies of occurrence in the demographic data. Furthermore, a 2 (gender of parents) x 2 (academic level) x 2 (competitive level) x 2 (sport typology) factorial analysis of variance (ANOVA) was applied to assess differences across the statement importance rankings included as the dependent variable. For each 80 ANOVA model, the magnitude of effect were measured whether the F-test was significant. The effect size (ES), partial Eta-squared [43,44] was used as the indicator of the degree of variability on the statement importance ranking as the dependent variable accounted for by the demographic data (e.g. gender of parents; academic level; competitive level; sport typology). Actually, partial Eta-squared is a coefficient not confined to a single independent variable. Therefore, the degree of variability on the dependent variable can be measured on the main effects as well as on the interactions simultaneously. [43,45,46].

For the concept mapping, the Ariadne software computed a binary symmetric similarity matrix per respondent and provided an aggregated matrix by merging the individual matrices, with high values indicating that many of the participants put the statements together in a group implying a conceptual similarity between statements [47]. This aggregated similarity matrix was transformed into a two-dimensional space. Thus, a stepwise analysis provided the clustering of statements (from 2 to 18) and the graphic representation of their origin. Finally, a spatial distribution (i.e., vertical and horizontal) of all clusters on the map mirrored the different themes deemed meaningful and identified by the participants.[30].

According to the literature on validity and reliability of concept mapping methodology [48], in the present study the external validity and internal validity have been considered. To mirror a conceptual model of the EMPATIA project [48], the external representational validity encompassed a systematic revision of the literature [26] and the assessment of coherence of the parents’ insights [23]. For the internal representational validity, the stress index was used as a goodness-of-fit indicator [49,50]. Specifically, values around 0.1 reflect a goodness-of-fit of the map to the original input (dissimilarity) matrix as routinely applied key diagnostic statistic in multidimensional scaling [33,48].

To verify the consistency of the sorting data of the concept mapping process, a group-belongingness via random assignment was used for identifying two split-half total reliabilities [48,51]. For each group, separate similarity matrix and maps, and Pearson correlation coefficients were computed, whereas Spearman-Brown correction was applied to obtain the split-half total matrix reliability (rSHT) [52]. The coordinates of the two-dimensional concept maps via metric multidimensional scaling on the sorted split-half data and after repeated Spearman-Brown corrections the split-half map reliability (rSHM) was computed. To assess the reliability estimates on the rating data (rRR), coefficients were computed as the average of the correlations between each respondents with Spearman-Brown Prophecy [51]. Following the quantitative analysis of the data and the graphic representation of the clusters and related statements, a qualitative analysis and interpretation was planned. The purpose of the qualitative analysis was to identify the least number of clusters that possess reasonable and agreed internal coherency and external theoretical distinction between the obtained clusters, to name the clusters and to reassign any statement, which may conceptually reside in another cluster. This qualitative and expert interpretation of the results is essential to optimise the knowledge translation and utilisation stemming from the research. The research output needs to be produced using constructs and terminology that are familiar to the end user, i.e. parents, athletes and policy makers. Thus, the EMPATIA research team participated in a dedicated workshop.

## Results

**Fig 1:**
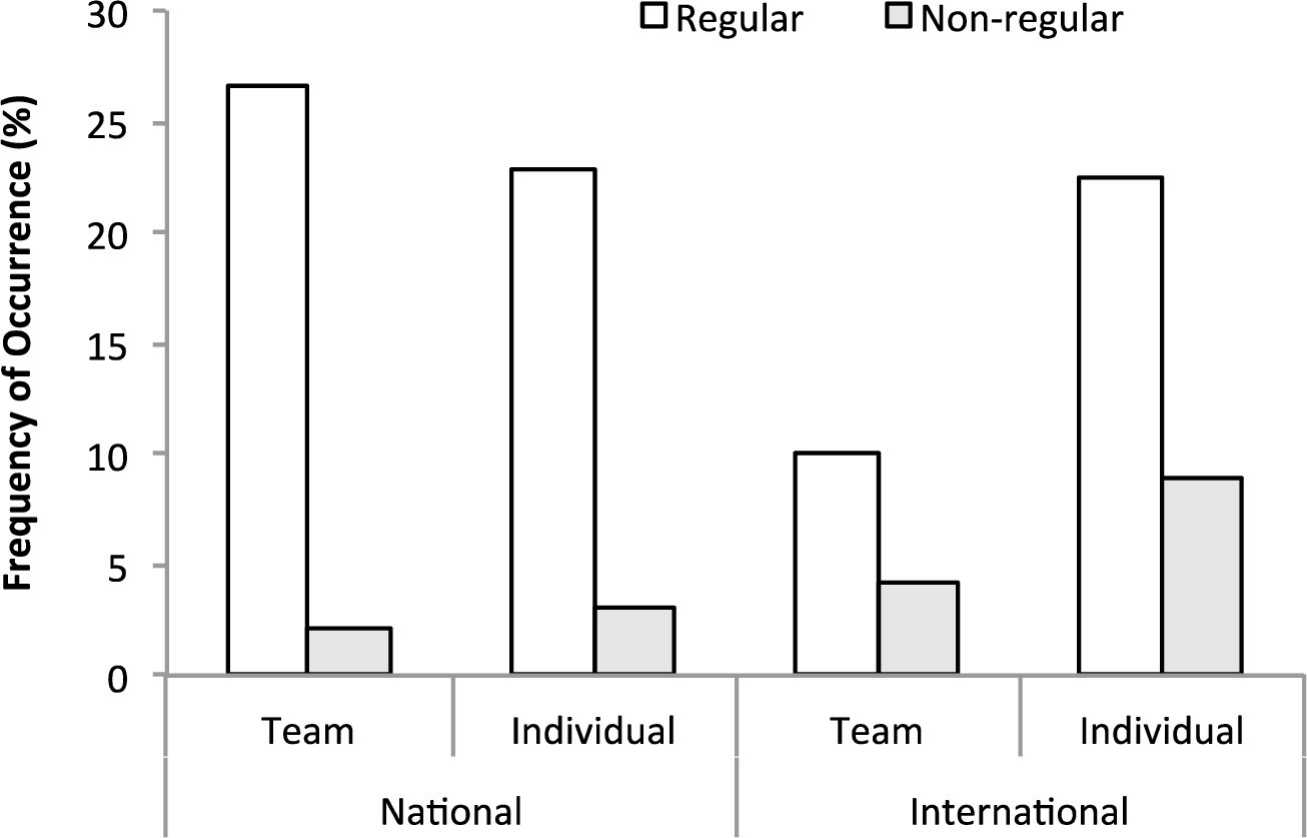
Frequency of occurrence (%) of regular (14–19 yr) and not-regular (>19 yr) high school student-athletes competing at national or international levels in individual or team sports.

Overall, 489 individuals (60% mothers, 40% fathers; France 24%, Ireland 11%, Italy 21%, Portugal 8% and Slovenia 36%) completed the online concept mapping exercise. However, a thorough examination of the responses excluded incomplete data responses or data duplication due to responses from both parents of an athlete. Thus, 337 responses were deemed acceptable for inclusion in the data analysis, encompassing parents of 162 female and 175 male student-athletes competing in individual (61%; athletics, biathlon, canoeing, competitive dance and fitness, diving, fencing, gymnastics, horse riding, ice skating, judo, karate, kayak, kickboxing, modern pentathlon, motorsports, mountain biking, rhythmic gymnastics, rowing, shooting, skiing, swimming, synchronized swimming, table tennis, taekwondo, tennis, thai boxing, triathlon, and weight lifting) and team (39%; American football, basketball, beach volleyball, camogie, football, handball, hockey, ice hockey, rugby, soccer, volleyball, and waterpolo) sports at national (53%) and international (47%) levels. Regarding the academic level of the athletes, 296 were enrolled at high school level, and 43 at university level. Regarding the age profile of the high school athletes only, 81.7% aged between 14–19 yrs (17.3±1.5 yrs) were regular-aged high school athletes, whereas 18.3% aged 20 years and older (22.7±1.7 yrs) were not-regular-aged students extending their time at high school. For high school athletes, differences for competition level and sport typology (Individual/Team) emerged (X^2^_(2, 4)_ = 18.53; p = 0.0003), with athletes competing at national level showing higher percentages of regular paths (60.3%) with respect to their international counterparts (39.7%), and athletes competing at international level in individual sports having a higher percentages of not-regular paths (68.4%) with respect to their team sport (31.6%) counterparts (Fig 1). Regarding living arrangements, 69.3% of the total sample lived at home with their parents, 23% lived in an athletic residence, and 7.8% lived away from parents.

No difference emerged between the gender of parents, and the competition and academic levels of the athletes. Conversely, a difference (F_(2, 244)_ = 4.9, p<0.001) emerged between the time spent by parents in supporting the athlete’ sport, and weekly sport training sessions logistics. The post hoc analysis maintained differences (p<0.001) between the sport support (7:40±6:30 hr:min) with respect to education (3:55±4:10 hr:min) and logistics (4:50±2:20 hr:min).

### Clustering and rating of statements

**Fig 2:**
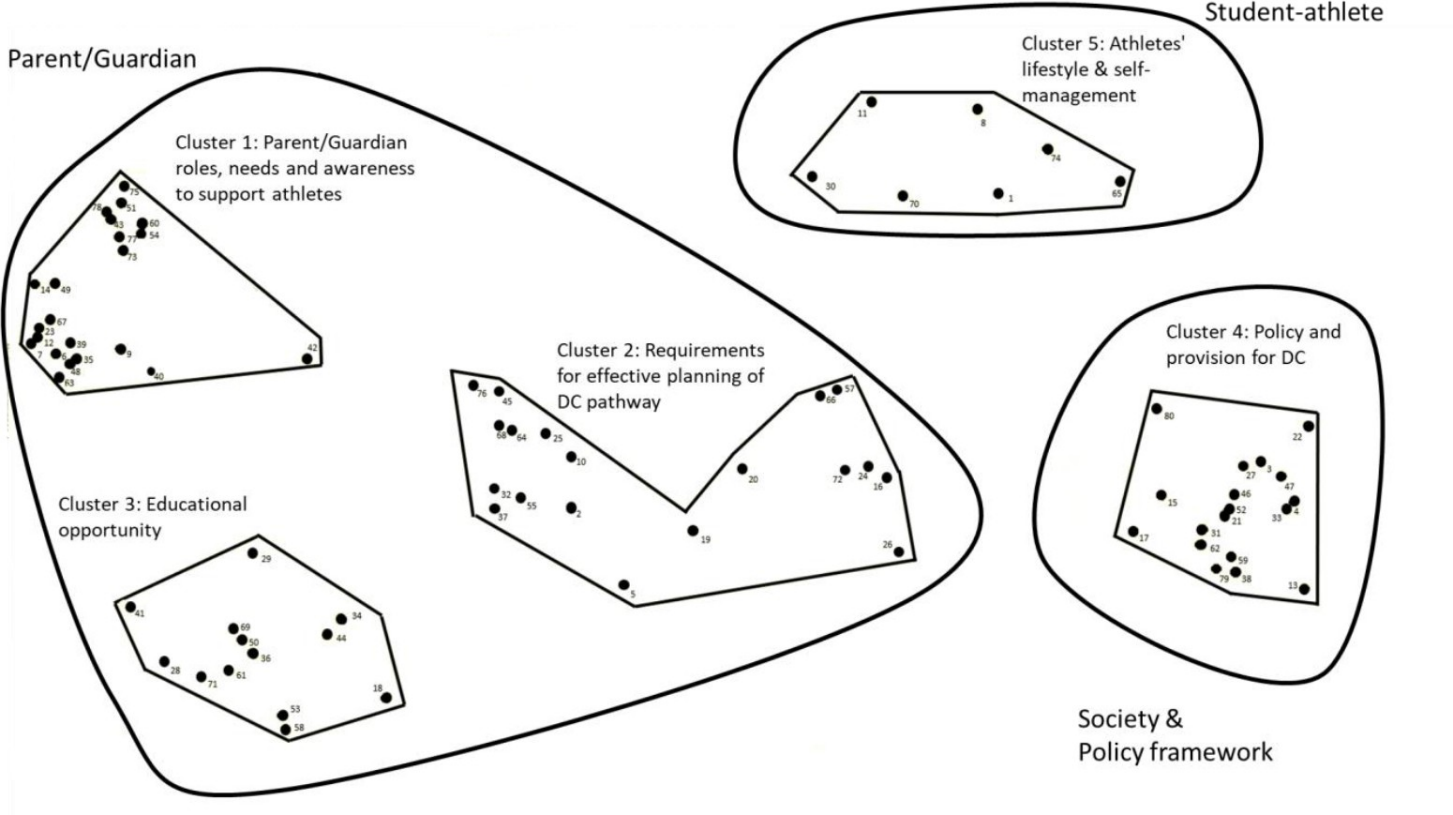
Five-cluster map within the three main areas Parent/Guardian (Cluster 1: Parent/Guardian roles, needs and awareness to support athletes; Cluster 2: Requirements for effective planning of dual career pathway; and Cluster 3: Educational opportunity), society and policy framework (Cluster 4: Policy and provision for dual career), and student-athlete (Cluster 5: Athletes’ lifestyle & self-management).

In merging the rating and sorting data the aggregated matrix the Ariadne software presents a frequency table where high mean values indicate high similarities among pairs of statements. To reduce and translate the multidimensionality of the matrix principal coordinate analysis (PcoA) was applied where the statements were plotted on a point map. The rating scores and graphic representation (i.e, point, map, cluster map) of the data informed all decisions regarding the identification of key statements and the statement clusters relevant to informing the EMPATIA framework. The stepwise analysis of the data provided 10 clusters, which had distinct origins and localised clustering of the 80 original statements mirroring the different themes deemed meaningful and identified by the participants. Four of the 10 clusters contained a single statement, two clusters contained between three and five statements and the four remaining clusters contained between seven and 21 statements. Through a qualitative interpretation, the EMPATIA research team concluded with a consensus around a five-cluster solution having a reasonable and agreed theoretical distinction between the clusters (Fig 2). The consensus process overcame the potential for spatial misrepresentation in the analysis by facilitated refinements based on human expertise, experience and the need for feasible solutions [33]. Finally, acceptable stress values (0.10 and 0.11) for the split halves, and high reliability for both input sorting data (rSHT = 0.980) and the two-dimensional coordinates of the multidimensional scaling results (rSHM = 0.995) emerged. For the rating data, high average rating reliability (rRR = 0.982) were found. Thus, the validity and reliability of the concept mapping was substantiated.

Subsequently, each cluster was assigned names, which represented the included statements and would be familiar and relevant to end users and stakeholders: 1) Parent/Guardian roles, needs and awareness to support athletes (health, well-being, sports, education), including 22 statements; 2) Requirements for effective planning of DC pathway, including 19 statements; 3) Educational opportunity, including 13 statements; 4) Policy and provision for DC, including 19 statements; and 5) Athletes’ lifestyle and self-management, including 7 statements (Table 2). It was possible to further refine this interpretation into three constructs, the first comprising 68% of all statements mainly related to the parents (e.g., ‘Parent’), the second comprising 24% of all statements mainly related to the DC socio-cultural-organizational statements (‘Society’) and the third comprising 9% of all statements mainly related to the student-athlete (‘Athlete’).

**Table 2:** Means ± standard deviations of the importance (pt) of identified statements by cluster in ascending order.

Number and statement by cluster	Importance (pt)
**Cluster 1: Parent/Guardian roles, needs and awareness to support athletes (health, well-being, sports, education)**	mean ± sd
75. Ability of parent/guardians to monitor the signs and symptoms of athlete wellbeing	4.2 ± 1.0
63. Information for parent/guardians on athlete medical needs and available supports	4.2 ± 1.0
39. Information for parent/guardians on athlete psychological needs and available supports	4.0 ± 1.0
6. Information for parent/guardians on athlete nutritional needs and available supports	4.0 ± 1.0
51. parent/guardian understanding/resolving athlete’s risky behaviors e.g. crises, alcohol & drug use, gambling	4.0 ± 1.1
48. More information for parent/guardian regarding rest, injury management and rehabilitation of the athlete	4.0 ± 1.0
78. Preparing parent/guardians to deal with emotional response of athlete to sport performance	4.0 ± 1.1
14. Parent/guardian understanding of their co-parenting role and support of the athlete.	3.8 ± 1.1
43. Parent/guardian awareness and understanding of sport as a way of life	3.8 ± 1.1
49. Parent/guardian skills to manage the family time commitment/logistics for athlete e.g. transport/time/roles	3.7 ± 1.2
60. Mother/female guardian perspective of importance of the athlete’s success in education & sport	3.6 ± 1.2
35. Communication skills/pathways for parent/guardian to assist the athlete sport career e.g. speaking to coach	3.6 ± 1.1
40. Information for parent/guardian on national procedure/service/opportunities for athlete in sport environment	3.6 ± 1.0
9. Support/guidelines for parent/guardian & athlete on conflict prevention /solution with sport environment	3.6 ± 1.1
54. Father/male guardian perspective of the importance of the athlete’s success in education & sport	3.5 ± 1.1
7. Parent/guardian basic knowledge of the various aspects/demands of the sport	3.5 ± 1.1
42. Best practice presentations by Dual Career athletes and parent/guardians of athletes	3.4 ± 1.1
67. Clarify parent/guardian role in supporting athlete in long-distance travel and intercultural experiences	3.4 ± 1.0
77. Clarify role of mother/female guardian for athlete in absence of supports/quality coaching	3.3 ± 1.2
73. Clarify role of father/male guardian for athlete in absence of supports/quality coaching	3.3 ± 1.2
23. Clarify the role of the parent/guardian in the sport environment	3.3 ± 1.1
12. Knowledge of how and when parent/guardians can join the athlete during training/competition	3.2 ± 1.2
**Cluster 2: Requirements for effective planning of Dual Career pathway**	mean ± sd
57. Access to quality coaching for athlete along the Dual Career pathway	4.5 ± 1.0
66. Dual Career awareness and support from the coach	4.3 ± 0.9
20. Information on potential to adapt sport schedule to accommodate demands of educational career	4.1 ± 1.0
19. Information on potential to adapt educational schedule to accommodate demands of sports career	4.1 ± 1.0
24. Advice for athlete on high school and university course selection	3.9 ± 1.1
16. Recognition of prior learning outcomes and sport experience for educational credits	3.9 ± 1.1
26. Develop list of Dual Career friendly schools and universities	3.9 ± 1.1
68. Knowledge for the parent/guardian to make a long term plan for the sport and education career of the athlete	3.8 ± 1.1
5. Information on the quality of schools from educational perspective	3.7 ± 1.1
55. Information for parent/guardians on financial cost/support in sport environment e.g. equipment/travel costs	3.7 ± 1.0
76. Good practice guidelines for parent/guardians to support the athletes	3.7 ± 1.0
45. More communication to parent/guardian from sport environment e.g. athlete progress/ranking/injury/training	3.6 ± 1.2
10. Communication skills/pathways for parent/guardians to assist athlete education career e.g. speaking to tutor	3.6 ± 1.1
72. Access to Dual Career help desk/contact person available in education environment	3.6 ± 1.1
37. Information for parent/guardian on financial management skills relevant to the student-athlete	3.4 ± 1.1
64. Clarify role of parent/guardian in the education environment e.g. homework support/negotiate for athlete	3.4 ± 1.1
32. Information for parent/guardians on selection procedures within sports	3.3 ± 1.1
2. Technology/social media knowledge for parent/guardians to access educational information relevant to athlete	3.2 ± 1.1
25. Technology/social media knowledge for parent/guardians to access sport information relevant to athlete	3.2 ± 1.1
**Cluster 3: Educational opportunity**	mean ± sd
28. Information for parent/guardians on relevance of education/degree for future opportunities of athletes	4.0± 1.0
41. Parent/guardian information on financial cost/support in education environment e.g. fees/housing/scholarships	3.7 ± 1.1
58. Parent/guardian information on national procedure/ service/opportunity for athlete in education environment	3.7 ± 1.1
61. Information for parent/guardian on educational supports for the athlete e.g. class notes/flexible deadlines	3.6 ± 1.1
50. parent/guardians feedback regarding educational progression at university (agreement of athlete required)	3.6 ± 1.1
71. Information for parent/guardians on how co-operation between sport and educational environments takes place	3.6 ± 1.1
29. Support/guidelines for parent/guardian & athlete on conflict prevention/solution with education environment	3.5 ± 1.2
44. Provide regular updates to parent/guardians on changes in policy, services and support for athletes	3.3 ± 1.1
53. Information for parent/guardians on international Dual Career policies/services available in education environment	3.3 ± 1.1
36. Information for parent/guardians on international Dual Career policies/services available in the sport environment	3.2 ± 1.2
34. E-Learning Opportunities: parent/guardian access to Dual Career social media networks, interactive forums & websites	3.2 ± 1.3
18. Dual Career educational brochure for parent/guardians which includes polices, opportunities, services etc	3.2 ± 1.1
69. Dual Career workshops for parent/guardians on education, sport & policy environments e.g.supports/best practice/law	3.1 ± 1.1
**Cluster 4: Policy and provision for Dual Career**	mean ± sd
59. Development of national policy to formally recognise student-athlete (Dual Career) status	4.2 ± 1.1
31. Provision of sport calendar to facilitate planning for educational career	4.1 ± 1.1
21. Policy to support athletes at end of sport career	4.0 ± 1.2
4. Increased awareness of the needs & circumstances of the athlete in the educational environment	4.0 ± 1.0
62. Provision of education calendar to facilitate planning for sport career	3.9 ± 1.1
38. Distance education provision for athletes e.g. at training centres /when travelling due to sport	3.9 ± 1.2
13. Sport partnerships with educational institutions to support Dual Career	3.9 ± 1.1
22. Mentoring for athlete in both sports and education environments	3.9 ± 1.1
15. Increased awareness of the needs & circumstances of the athlete in the educational environment	3.8 ± 1.0
79. Development of international policy to formally recognise student-athlete (Dual Career) status	3.8 ± 1.1
33. Sport partnerships with industry/business to support Dual Career	3.7 ± 1.1
3. Dual Career support providers in each sport federation	3.6 ± 1.2
80. Access to Dual Career help desk/contact person available in sport environment	3.6 ± 1.1
47. Identification of successful student-athletes who can act as ambassadors for dual career	3.4 ± 1.1
46. Access to/appointment of national Dual Career coordinator to support the needs of athletes	3.4 ± 1.2
52. Inclusion of non-elite/talented athletes in Dual Career athlete legislation	3.3 ± 1.2
17. Knowledge and exemplars of national and international Dual Career best practice	3.3 ± 1.0
27. Utilising sport clubs as hubs for Dual Career information sharing and updates	3.3 ± 1.1
56. Representation of parent/guardians at sport administration/federation levels	3.1 ± 1.2
**Cluster 5: Athletes’ lifestyle & self-management**	mean ± sd
1. Ability of athlete to effectively manage Dual Career time e.g. school, study, sport	4.5 ± 0.9
11. Ongoing evaluation of athlete mental health and wellbeing	4.3 ± 1.0
74. Ability of athlete to effectively manage independent living e.g. cooking, cleaning, resting	4.0 ± 1.1
30. Setting of realistic & achievable goals for parent/guardians & athletes in sport and educational environments	3.9 ± 1.1
65. Athlete valuing their Dual Career status as a way of life	4.0 ± 1.1
8. Ongoing evaluation of athlete life style	3.7 ± 1.1
70. Understanding impact of Dual Career on socialisation & social interactions of athlete e.g. siblings/peers	3.5 ± 1.1

For the 80 statements, the ranking of importance ranged between 3.1±1.2 and 4.5±0.9 pt, with the means of the statements ranks included in the clusters ranging from 3.46 to 3.98 pt. The proportion of statements rated ≥4.0 pt. was 25%, these were considered to have greater relevance for planning a DC parenting education programme. The highest distribution was in cluster 1 (n = 7) and the lowest in cluster 3 (n = 1).

The factorial ANOVA was conducted on the importance rankings of the 80 statements as the dependent variable and four demographic variables (*gender of parents*, *academic level*, *competitive level*, *sport typology*) as independent variables. In 29 cases, significant main effects on one of those variables emerged. The grouped category of means of the independent variables revealed certain pattern (Table 3). As the higher and lower values are separated and belong to the same category of the independent variables.

**Table 3:** Interactions between dependent variables.

Number and statement	Variables				
	**Gender of parents**				
	Father	Mother	F	p	ES
24. Advice for athlete on high school and university course selection	3.8±1.1	4.0±1.1	5.89	0.016#	0.02
40. Information for parent/guardian on national procedure/service/opportunities for athlete in sport environment	3.3±1.1	3.7±1.0	8.28	0.004	0.03
41. Parent/guardian information on financial cost/support in education environment e. g. fees/housing/scholarships	3.5±1.1	3.9±1.0	4.44	0.036	0.01
43. Parent/guardian awareness and understanding of sport as a way of life	3.7±1.2	3.9±1.1	4.63	0.032	0.01
49. Parent/guardian skills to manage the family time commitment/logistics for athlete e. g. transport/time/roles	3.6±1.2	3.8±1.2	3.96	0.047	0.01
61. Information for parent/guardian on educational supports for the athlete e.g. class notes/flexible deadlines	3.4±1.1	3.8±1.1	9.35	0.002	0.03
62. Provision of education calendar to facilitate planning for sport career	3.8±1.2	4.0±1.1	4.71	0.031#	0.01
67. Clarify parent/guardian role in supporting athlete in long-distance travel and intercultural experiences	3.1±1.0	3.5±1.0	6.93	0.009	0.02
68. Knowledge for the parent/guardian to make a long term plan for the sport and education career of the athlete	3.6±1.1	4.0±1.1	7.31	0.007	0.02
74. Ability of athlete to effectively manage independent living e.g. cooking, cleaning, resting	3.9±1.1	4.1±1.0	4.49	0.035*	0.01
	**Educational level of athletes**				
	High school	University	F	p	ES
2. Technology/social media knowledge for parents/guardians to access educational information relevant to athlete	3.3±1.1	2.9±1.0	3.98	0.047	0.01
7. Parent/guardian basic knowledge of the various aspects/demands of the sport	3.5±1.1	3.3±1.3	5.34	0.021*	0.02
29. Support/guidelines for parent/guardian & athlete on conflict prevention/solution with education environment	3.6±1.1	3.0±1.3	10.18	0.002	0.03
35. Communication skills/pathways for parent/guardian to assist the athlete sport career e.g. speaking to coach	3.6±1.1	3.3±1.3	5.78	0.017#	0.02
48. More information for parent/guardian regarding rest, injury management and rehabilitation of the athlete	4.0±1.0	3.7±1.1	6.09	0.014	0.02
54. Father/male guardian perspective of the importance of the athlete’s success in education & sport	3.6±1.1	3.1±1.2	4.65	0.032#	0.01
60. Mother/female guardian perspective of importance of the athlete’s success in education & sport	3.7±1.2	3.3±1.1	6.68	0.010#	0.02
63. Information for parents/guardians on athlete medical needs and available supports	4.2±1.0	4.0±1.0	6.97	0.008#*	0.02
	**Competition level of athletes**				
	National	International	F	P	ES
3. Dual Career support providers in each sport federation	3.7±1.1	3.6±1.2	4.95	0.027#	0.02
17. Knowledge and exemplars of national and international Dual Career best practice.	3.4±1.0	3.3±1.1	4.42	0.036#	0.01
18. Dual Career educational brochure for parents/guardians which includes polices, opportunities, services etc.	3.3±1.1	3.1±1.1	4.74	0.030#	0.01
36. Information for parents/guardians on international Dual Career policies/services available in the sport environment	3.3±1.1	3.2±1.2	7.74	0.006#*	0.02
46. Access to/appointment of national Dual Career coordinator to support the needs of athletes	3.5±1.1	3.3±1.3	4.07	0.044	0.01
47. Identification of successful student-athletes who can act as ambassadors for dual career	3.5±1.1	3.3±1.1	6.51	0.011#	0.02
73. Clarify role of father/male guardian for athlete in absence of supports/quality coaching	3.1±1.2	3.5±1.2	5.98	0.015	0.02
	Sport typology of athletes				
	Individual	Team	F	P	ES
25. Technology/social media knowledge for Parents/guardians to access sport information relevant to athlete	3.3±1.1	3.1±1.1	4.13	0.043	0.01
32. Information for parents/guardians on selection procedures within sports	3.4±1.1	3.3±1.0	4.16	0.042	0.01
45. More communication to parent/guardian from sport environment e.g. athlete progress/ranking/injury/training	3.7±1.1	3.6±1.2	6.11	0.014#	0.02
55. Information for parents/guardians on financial cost/support in sport environment e.g. equipment/travel costs	3.9±1.0	3.5±1.1	5.00	0.026	0.02

# = two-way interaction

* = three-way interaction.

Interactions among the different demographic categories were also examined (Table 4) Significant interactions were found. The occurrence of the association were present mainly in two-way interaction with either the *academic level* or the *gender of parents*. Overall, 23 significant two -way interactions on 19 different statement importance rankings emerged. Furthermore, 12 significant three -way interactions were present on 9 rankings and, in the most complex setup, two four-way interactions were statistically significant on two rankings. These interactions corroborated and amplified the unfolded tendency of segmented categories of the independent variables associated with the main effects.

**Table 4:** Interactions between dependent variables.

Number and statement	Variables				
	**Gender of parents**				
	Father	Mother	F	p	ES
24. Advice for athlete on high school and university course selection	3.8±1.1	4.0±1.1	5.89	0.016#	0.02
40. Information for parent/guardian on national procedure/service/opportunities for athlete in sport environment	3.3±1.1	3.7±1.0	8.28	0.004	0.03
41. Parent/guardian information on financial cost/support in education environment e. g. fees/housing/scholarships	3.5±1.1	3.9±1.0	4.44	0.036	0.01
43. Parent/guardian awareness and understanding of sport as a way of life	3.7±1.2	3.9±1.1	4.63	0.032	0.01
49. Parent/guardian skills to manage the family time commitment/logistics for athlete e. g. transport/time/roles	3.6±1.2	3.8±1.2	3.96	0.047	0.01
61. Information for parent/guardian on educational supports for the athlete e.g. class notes/flexible deadlines	3.4±1.1	3.8±1.1	9.35	0.002	0.03
62. Provision of education calendar to facilitate planning for sport career	3.8±1.2	4.0±1.1	4.71	0.031#	0.01
67. Clarify parent/guardian role in supporting athlete in long-distance travel and intercultural experiences	3.1±1.0	3.5±1.0	6.93	0.009	0.02
68. Knowledge for the parent/guardian to make a long term plan for the sport and education career of the athlete	3.6±1.1	4.0±1.1	7.31	0.007	0.02
74. Ability of athlete to effectively manage independent living e.g. cooking, cleaning, resting	3.9±1.1	4.1±1.0	4.49	0.035*	0.01
	**Educational level of athetes**				
	High school	University	F	p	ES
2. Technology/social media knowledge for parents/guardians to access educational information relevant to athlete	3.3±1.1	2.9±1.0	3.98	0.047	0.01
7. Parent/guardian basic knowledge of the various aspects/demands of the sport	3.5±1.1	3.3±1.3	5.34	0.021*	0.02
29. Support/guidelines for parent/guardian & athlete on conflict prevention/solution with education environment	3.6±1.1	3.0±1.3	10.18	0.002	0.03
35. Communication skills/pathways for parent/guardian to assist the athlete sport career e.g. speaking to coach	3.6±1.1	3.3±1.3	5.78	0.017#	0.02
48. More information for parent/guardian regarding rest, injury management and rehabilitation of the athlete	4.0±1.0	3.7±1.1	6.09	0.014	0.02
54. Father/male guardian perspective of the importance of the athlete’s success in education & sport	3.6±1.1	3.1±1.2	4.65	0.032#	0.01
60. Mother/female guardian perspective of importance of the athlete’s success in education & sport	3.7±1.2	3.3±1.1	6.68	0.010#	0.02
63. Information for parents/guardians on athlete medical needs and available supports	4.2±1.0	4.0±1.0	6.97	0.008#*	0.02
	**Competiton level of athetes**				
	National	Inter-national	F	P	ES
3. Dual Career support providers in each sport federation	3.7±1.1	3.6±1.2	4.95	0.027#	0.02
17. Knowledge and exemplars of national and international Dual Career best practice.	3.4±1.0	3.3±1.1	4.42	0.036#	0.01
18. Dual Career educational brochure for parents/guardians which includes polices, opportunities, services etc.	3.3±1.1	3.1±1.1	4.74	0.030#	0.01
36. Information for parents/guardians on international Dual Career policies/services available in the sport environment	3.3±1.1	3.2±1.2	7.74	0.006#*	0.02
46. Access to/appointment of national Dual Career coordinator to support the needs of athletes	3.5±1.1	3.3±1.3	4.07	0.044	0.01
47. Identification of successful student-athletes who can act as ambassadors for dual career	3.5±1.1	3.3±1.1	6.51	0.011#	0.02
73. Clarify role of father/male guardian for athlete in absence of supports/quality coaching	3.1±1.2	3.5±1.2	5.98	0.015	0.02
	Sport typology of athletes				
	Indivi-dual	Team	F	p	ES
25. Technology/social media knowledge for Parents/guardians to access sport information relevant to athlete	3.3±1.1	3.1±1.1	4.13	0.043	0.01
32. Information for parents/guardians on selection procedures within sports	3.4±1.1	3.3±1.0	4.16	0.042	0.01
45. More communication to parent/guardian from sport environment e.g. athlete progress/ranking/injury/training	3.7±1.1	3.6±1.2	6.11	0.014#	0.02
55. Information for parents/guardians on financial cost/support in sport environment e.g. equipment/travel costs	3.9±1.0	3.5±1.1	5.00	0.026	0.02

# = two-way interaction

* = three-way interaction.

## Discussion

The EMPATIA collaborative partnership [590437-EPP-1-2017-1-SI-SPO-SCP] aimed to develop a framework of parenting DC athletes based on the parents’ view [29]. This study capitalized the combined state of the art knowledge [26] and the experience of a wide representation of European parents of talented and elite student-athletes competing in different sports [23], adding the novel empirical approach to define a European framework of parenting DC athletes based on the parents’ rating and clustering the potential statements of parenting support of student-athletes. According to the literature [30], one of the crucial aspects of concept mapping is the selection of participants, which should include a broad heterogeneous participation to provide a wide variety of viewpoints contributing to a conceptual framework generalizable to the population of interest. Based on the consistent data collection including a large and various sample of parents representing different Member States, sports, and academic levels, the EMPATIA framework epitomizes a rich account of parenting DC athletes. Therefore, the high validity and reliability of the present findings could be framed as potential lessons to optimize our current understanding of the parents’ needs to support student-athletes and to provide significant guidance to the future DC research agenda and subsequent translation into policies [11]. Furthermore, the analysis based on the gender-related parental support of DC athletes and the academic and sport commitment of the student-athletes allowed to further identify relevant aspects of parental supportive role. This unique approach allowed highlighting specific aspects, which might help differentiating DC approaches to parental support also in relation to the sport, the competitive level and the academic levels of the student-athletes.

The distribution of statements within the five clusters organized in three main areas provides insight into the collective vision of parents regarding how to address parenting DC athletes. Similarly, to the third-order constructs of elite athletes’ experiences in DC development [53], the area “Parent” describes the micro-level, whereas the area “Athlete” refers to the inter-individual meso level, and the area “Society” concerns the macro and policy levels, respectively [11,12].

Resonating previous research on parenting DC athletes [18,26], the ‘Parent’ theme infers that the individual is central in the support of the student-athlete. The area ‘Parent’ encompasses aspects related to the parental role (cluster 1) to the actual support (cluster 2) and the means of education (cluster 3), which informed three main aspects (e.g., Who, How, and What, respectively) of the EMPATIA educational programme for parents of DC athletes (https://edu.empatiasport.eu/eng/). The area ‘Society’ comprises a cluster urging Governments, educational institutions and sport bodies to create and strengthen supporting DC environments, which could enable successful academic and sport paths, thus echoing the plead for attention to the role of policy and the environment in promoting DC of European athletes [11,12,14], also contributing to the development of a European DC culture [15,16]. According to the literature [54], applied implications to enhance parental support should consider families in the sport and education decision bodies to enhance listening, questioning, and negotiation, and to experience changes in their social networks. This area informed a fourth aspect of a DC educational programme (e.g., Why). Finally, the third area ‘Athlete’ comprises a cluster related to the individual responsibility of pursuing DC paths, which relies on the micro level of the single athletes according to their sex, age, practiced sport, previous international athletic experience, and academic level [18,55–59]. Indeed, student-athletes not only have to be dedicated, resilient, and determined, but also need to develop high autonomy and responsibility to optimise their capability to plan in advance, to prioritise what needs to be done, and to and use time efficiently [55–57,59–61]. Thus, the parents participating in the present study, not only focused on their perspectives, roles, and opportunities for parental support of DC athletes, but also valued the societal role and the athlete’s autonomy [28,54,62,63].

Within the ‘Parent’ area, the 28% of the statements captured in the Parent/Guardian roles, needs and awareness to support athletes (health, well-being, sports, education), the findings of this study confirmed the parental concern of the child’s health and well-being and their capability to detect early signs for risks prevention, independently from their gender-related parenting role. The present findings support the independent and interactive contributions of mothers and fathers reported in the literature [26]. Despite some authors claimed that fathers are marginally involved in the daily organization schedules of the athlete [58], in this study the no gender-related difference emerged for parental participation, as well as for the time of parental support of the talented and elite athletes in sport (~8 hr), education (~5 hr) and logistics (~5 hr). Thus, it is possible to speculate that talented and elite student-athletes have a full support from their parents. Conversely, mothers resulted significantly more concerned with respect to fathers regarding their awareness and understanding of sport as a way of life, how to manage the family in relation to the athlete’s needs, the opportunities and services offered at sport level, and their role during long-distance travelling of the athlete, indicating that mothers’ perceptions towards parental information regarding the sport environment is crucial to support adequately the athlete. This gender-related difference could be associated to the dominant role of fathers in shaping their children’s sport experiences and the male relevant role in the sports domain [64,65]. In considering that parents with a sport-specific cultural capital could help their children to persist and succeed in their chosen sport [66], positive results could be expected from the involvement in the sporting community of women not directly involved in sport but supporting their talented and elite progeny [14].

With respect to their counterparts parenting university athletes, parents of high school athletes rated higher the importance of probabilistic opportunities for the athlete’s academic and sport success, the need of sport-specific knowledge and communication with sport staff, as well as information of health care of the athletes. Indeed, during high school academic and athletic demands often intensify simultaneously, which need the parents’ capability to detect burnout symptoms leading to risk of school and/or sport dropouts [67]. In line with the recommendations of the literature [68], parents are aware that effective communication with coaches, teachers, and health-care professionals is necessary to achieve a thorough understanding of the current demands and resources of the adolescents student-athlete, thus facilitating the early detection and the prevention of dropout risks. This aspect could be particularly relevant for the parents of female student-athletes, whose aspirations to become elite athletes could decline over time [66]. Actually, to balance collaboration between high schools and sports clubs and to favour opportunities for student-athletes to develop their athletic talents while also developing non-athletic career aspirations, some Member States have formal sport schools for talented and elite athletes, whereas others provide special programmes tailored to student-athletes [12]. Furthermore, sports with established national and international leagues (e.g., football, basketball, handball, rugby) could have formal educational opportunities for athletes enrolled in their respective sport academies [69,70]. In general, high schools include a representation of parents in their decision bodies and establish formal and informal communication between teachers and families, whereas sport academies should implement the communication flow with parents. Undoubtedly, formal agreements between sports bodies and educational institutions could be envisaged to provide parents comprehensive information on the academic and athletic progress of their child [54]. Despite the statistical difference, also the parents of athletes enrolled at tertiary levels considered relevant these aspects, indicating that DC support services should develop and make available resources and programs designed to enhance parental communication for optimizing the athlete’s wellbeing and well-functioning.

The differences emerging for competition level highlighted the highest concern of parents of athletes competing at a national level on DC best practices and support provision. In the literature [58], athletes not competing at the highest level tend to consider education a valuable alternative to their transition to the labour market, thus, it is possible to speculate that their parents’ need of information on DC at national and international levels mirrors their desire to equip their child with the possibility of building a personal education and a sport progression toward a prospect peak performance [62,71,72]. In fact, in a globalized mobility strongly characterizes the personal development of European youth through education and a relevant opportunity for athletic progression [1,73] Conversely, parents of international student-athletes showed a highest concern towards a better coaching, probably due to a wide conceptualisation of quality coaching as related to the athlete’s outcomes rating large significant business revenues and sponsorships [74,75], which relief the parents from a financial burden for equipment, travelling, and professional services. Therefore, parents of student-athletes competing in individual sport might need a constant monitoring of the progression and needs of their child.

## Conclusions

The uniqueness of the present study lies in the efforts of a consistent number of European parents of student-athletes who contributed to uncover the multi-level relationships between aspects of parenting DC athletes applicable to individuals, to the sport and academic contexts and to the society. In synergizing a range of knowledge, capacities, activities, and actions, the EMPATIA framework presents a valuable conceptual map, which generated information for the EMPATIA multilingual (e.g., English, French, Italian, Portuguese, and Slovenian), educational programme on parenting DC athletes (https://edu.empatiasport.eu/eng/).

The practical relevance of the EMPATIA framework subsumes the resulting theoretical evidence on parenting student-athletes as a sound basis for practical decision-making and urges stakeholders from the academic and sport athlete’s entourages to coordinate their efforts in bridging existing DC gaps, establishing alliances also with the parents by including their representatives in the decision-bodies. Thus, the results of this initiative could potentially contribute to the development of a DC practices at local, national, and Pan-European level, for an effective contribution to the European DC discourse [11,16].

The current study indicated pivotal similarities of the parents related to their perceptions on DC of their children. The findings suggest that parents share the principle in terms of the most relevant aspects. Nevertheless, several significant differences among these aspects emerged, including additional variables (gender of parents, and academic level, competition level and the type of sport of the athletes). These patterns suggest that behind the overall figures and conclusions the internal structure contains substantial patterns to unfold. Therefore, further research should conduct to scrutinize the structure and its content.

## Supporting information

S1 Data Background questionnaire responses from participants including demographical data. (XLSX)

S2 Data Participant’s sorting data of concept mapping analysis. (XLSX)

S3 Data Participant’s rating data of concept mapping analysis. (XLSX)

## References

[1] Council of the European Union. Resolution of the Council and of the Representatives of the Governments of the Member States, meeting within the Council, on the European Union Work Plan for Sport. 2017. Available from: https://eur-lex.europa.eu/legal-content/EN/TXT/HTML/?uri=CELEX:42017Y0615(01)&from=EN.

[2] Council of the European Union. Conclusions of the Council and of the Representatives of the Governments of the Member States, Meeting within the Council, on Dual Careers for Athletes. 2013. Available from: https://eur-lex.europa.eu/legal-content/EN/TXT/?uri=celex%3A52013XG0614%2803%29.

[3] Council of the European Union. Resolution of the Council and of the Representatives of the Governments of the Member States, meeting within the Council, on a European Union Work Plan for Sport for 2011–2014. 2011. Available from: https://eur-lex.europa.eu/legal-content/HR/TXT/?uri=CELEX:42011Y0601(01).

[4] European Commission. EACEA—Education, Audiovisual and Culture Executive Agency (Sport Chapter in the Erasmus+ Programme). (2020). Available from: https://eacea.ec.europa.eu/erasmus-plus/actions/sport_en.

[5] European Commission. White Paper on Sport. (2007) Available from: https://eur-lex.europa.eu/legal-content/EN/TXT/HTML/?uri=CELEX:52007DC0391&from=EN.

[6] United Nations. Universal Declaration of Human Rights.1948. Available from: https://www.un.org/en/universal-declaration-human-rights/.

[7] United Nations. Convention on the Rights of the Child.1989. Available from: https://treaties.un.org/pages/ViewDetails.aspx?src=IND&mtdsg_no=IV-11&chapter=4&lang=en.

[8] De Brandt K, Wylleman P, Torregrossa M, Schipper-Van Veldhoven N, Minelli D, Defruyt S, et al. Exploring the factor structure of the dual career competency questionnaire for athletes in european pupil- and student-athletes. 2018; 1–18. 10.1080/1612197X.2018.1511619.

[9] Henry I. Athlete Development, Athlete Rights and Athlete Welfare: A European Union Perspective. The International Journal of the History of Sport. 2013; 30(4):356–73. 10.1080/09523367.2013.765721.

[10] Aquilina D, Henry I. Elite athletes and university education in Europe: A review of policy and practice in higher education in the European Union Member States. International Journal of Sport Policy and Politics. 2010;2(1):25–47. 10.1080/19406941003634024.

[11] Capranica L, Guidotti F. Qualifications/Dual Careers in Sports. 2016. Available from: https://www.europarl.europa.eu/thinktank/en/document.html?reference=IPOL_STU(2016)573416.

[12] European Commission. Directorate General for Education and Culture., Amsterdam University of Applied Sciences., Birch Consultants., Talented Athlete Scholarship Scheme., Vrije Universiteit Brussel. Study on the minimum quality requirements for dual career services: Final report. LU: Publications Office; 2016. Available from: https://data.europa.eu/doi/10.2766/345818.

[13] Stambulova NB, Ryba TV, Henriksen K. Career development and transitions of athletes: The international society of sport psychology position stand revisited. International Journal of Sport and Exercise Psychology. 2020;1–27. 10.1080/1612197X.2020.1737836.

[14] European Commission. Directorate General for Education and Culture. EU guidelines on dual careers of athletes: Recommended policy actions in support of dual careers in high performance sport. 2013. Available from: https://data.europa.eu/doi/10.2766/52683.

[15] Guidotti F, Cortis C, Capranica L. Dual career of European student-athletes: a systematic literature review. Kinesiologia Slovenica. 2015;21(3):5–20.

[16] Stambulova NB, Wylleman P. Psychology of athletes’ dual careers: A state-of-the-art critical review of the European discourse. Psychology of Sport and Exercise. 2019;42:74–88. 10.1016/j.psychsport.2018.11.013.

[17] Wylleman P, Lavallee D. A developmental perspective on transitions faced by athletes. Developmental sport and exercise psychology: a lifespan perspective. 2004;503–523.

[18] Condello G, Capranica L, Doupona M, Varga K, Burk V. Dual-career through the elite university student-athletes’ lenses: The international FISU-EAS survey. PLoS ONE. 2019; 14(10): e0223278. doi: 10.1371/journal.pone.0223278 31577823 PMC6774511

[19] Wendling E, Kellison TB, Sagas M. A conceptual examination of college athletes’ role conflict through the lens of conservation of resources theory. Quest. 2018;70(1):28–47. 10.1080/00336297.2017.1333437.

[20] Bean C, Fortier M, Post C, Chima K. Understanding how organized youth sport may be harming individual players within the family unit: A literature review. IJERPH. 2014;11(10):10226–10268. doi: 10.3390/ijerph111010226 25275889 PMC4210977

[21] Clarke NJ, Harwood CG. Parenting experiences in elite youth football: A phenomenological study. Psychology of Sport and Exercise. 2014;15(5):528–537. 10.1080/10413200.2016.1194909.

[22] Dorsch TE, King MQ, Dunn CR, Osai KV, Tulane S. The impact of evidence-based parent education in Organized Youth Sport: A Pilot Study. Journal of Applied Sport Psychology. 2017;29(2):199–214. 10.1080/10413200.2016.1194909.

[23] Gjaka M, Tessitore A, Blondel L, Burlot F, Costa R, Debois N, et al. Understanding the Educational Needs of Dual Career Parenting: The Parents’ View. 2020. [Manuscript submitted for publication].

[24] Harwood C, Drew A, Knight CJ. Parental stressors in professional youth football academies: a qualitative investigation of specialising stage parents. Qualitative Research in Sport and Exercise. 2010;2(1):39–55. 10.1080/19398440903510152.

[25] Harwood CG, Knight CJ. Parenting in youth sport: A position paper on parenting expertise. Psychology of Sport and Exercise. 2015;16:24–35. http://doi.apa.org/getdoi.cfm?doi=10.1037/spy0000063.

[26] Tessitore A, Capranica L, Pesce C, De Bois N, Gjaka M, Warrington G, et al. Parents about parenting dual career athletes: A systematic literature review. Psychology of Sport and Exercise. 2020;101833. doi: 10.1016/j.psychsport.2020.101833 33110396 PMC7581327

[27] Harwood C, Knight C. Understanding parental stressors: An investigation of British tennis-parents. Journal of Sports Sciences. 2009;27(4):339–351. doi: 10.1080/02640410802603871 19191064

[28] Thrower SN, Harwood CG, Spray CM. Educating and supporting tennis parents: A grounded theory of parents’ needs during childhood and early adolescence. Sport, Exercise, and Performance Psychology. 2016;5(2):107–124. 10.1037/spy0000054.

[29] Capranica L, MacDonncha C, Blondel L, Bozzano E, Burlot F, Costa R, et al. Towards the construction of an educational model for dual career parenting: the EMPATIA project. Kinesiologia Slovenica. 2018;24(3):19–30.

[30] Trochim WMK. An introduction to concept mapping for planning and evaluation. Evaluation and Program Planning. 1989;12(1):1–16. 10.1016/0149-7189(89)90016-5.

[31] Trochim WMK. Concept Mapping: Soft Science or Hard Art? Evaluation and Program Planning. 1989;12(1):87–110. 10.1016/0149-7189(89)90027-X.

[32] Jackson KM, Trochim WMK. Concept mapping as an alternative approach for the analysis of open-ended survey responses. Organizational Research Methods. 2002;5(4):307–336. 10.1177/109442802237114.

[33] Kane M, Trochim W. Concept mapping for planning and evaluation. Thousand Oaks: SAGE Publications, Inc.; 2007 Available from: http://methods.sagepub.com/book/concept-mapping-for-planning-and-evaluation.

[34] Cousineau TM, Franko DL, Ciccazzo M, Goldstein M, Rosenthal E. Web-based nutrition education for college students: Is it feasible? Evaluation and Program Planning. 2006;29(1):23–33. doi: 10.1016/j.evalprogplan.2005.04.018 21494421 PMC3074521

[35] Cousineau T, Houle B, Bromberg J, Fernandez KC, Kling WC. A pilot study of an online workplace nutrition program: The value of participant input in program development. Journal of Nutrition Education and Behavior. 2008;40(3):160–167. doi: 10.1016/j.jneb.2007.04.376 18457784 PMC2993184

[36] Chastin SFM, De Craemer M, Lien N, Bernaards C, Buck C, et al. The SOS-framework (Systems of Sedentary behaviours): An international transdisciplinary consensus framework for the study of determinants, research priorities and policy on sedentary behaviour across the life course: a DEDIPAC-study. International Journal of Behavioral Nutrition and Physical Activity. 2016 Dec;13(1):83. 10.1186/s12966-016-0409-3. PMC4947275 27421750

[37] Condello G, Ling FCM, Bianco A, Chastin S, Cardon G, et al. Using concept mapping in the development of the EU-PAD framework (EUropean-Physical Activity Determinants across the life course): a DEDIPAC-study. BMC Public Health. 2016;16(1):1145. doi: 10.1186/s12889-016-3800-8 27825370 PMC5101801

[38] Visek AJ, Achrati SM, Mannix HM, McDonnell K, Harris BS, DiPietro L. The fun integration theory: Toward sustaining children and adolescents sport participation. Journal of Physical Activity and Health. 2015;12(3):424–433. doi: 10.1123/jpah.2013-0180 24770788 PMC4201634

[39] Donaldson A, Callaghan A, Bizzini M, Jowett A, Keyzer P, Nicholson M. A concept mapping approach to identifying the barriers to implementing an evidence-based sports injury prevention programme. Injury Prevention. 2019;25(4):244–251. doi: 10.1136/injuryprev-2017-042639 29353246

[40] Van Slingerland KJ, Durand-Bush N, Kenttä G. Collaboratively designing the Canadian Centre for Mental Health and Sport (CCMHS) using group concept mapping. Journal of Applied Sport Psychology. 2020;1–25. 10.1080/10413200.2019.1704938.

[41] Capranica L, Foerster J, Keldorf O, Leseur V, Vandewalle P, Mojca DT, et al. The European Athlete as Student Network (“EAS”): prioritising dual career of European student-athletes. Kinesiologia Slovenica. 2015;21(2):5–10.

[42] Deutskens E, de Ruyter K, Wetzels M, Oosterveld P. Response rate and response quality of internet-based surveys: An experimental study. Marketing Letters. 2004;15(1):21–36. 10.1023/B:MARK.0000021968.86465.00.

[43] Cohen J. Eta-Squared and Partial Eta-Squared in Fixed Factor ANOVA Designs. Educational and Psychological Measurement. 1973;(1):107–112. 10.1177/001316447303300111.

[44] Cohen J. A power primer. Psychological Bulletin. 1992;112(1):155–159. doi: 10.1037//0033-2909.112.1.155 19565683

[45] Keppel G, Wickens TD. Design and Analysis: A Researcher’s Handbook. Pearson Prentice Hall; 2004.

[46] Lakens D. Calculating and reporting effect sizes to facilitate cumulative science: A practical primer for t-tests and ANOVAs. Frontiers in Psychology. 2013; 4. Available from: doi: 10.3389/fpsyg.2013.00863 24324449 PMC3840331

[47] Manual ARIADNE 3.0. 2015. Available from: http://www.minds21.org/images_public/manual%20%20ARIADNE%203.0%20%20april%202015.pdf.

[48] Rosas SR, Kane M. Quality and rigor of the concept mapping methodology: A pooled study analysis. Evaluation and Program Planning. 2012 May;35(2):236–45. doi: 10.1016/j.evalprogplan.2011.10.003 22221889

[49] Kruskal JB. Multidimensional scaling by optimizing goodness of fit to a nonmetric hypothesis. Psychometrika. 1964 Mar;29(1):1–27.

[50] Kruskal J, Wish M. Multidimensional Scaling [Internet]. 2455 Teller Road, Thousand Oaks California 91320 United States of America: SAGE Publications, Inc.; 1978 [cited 2020 Nov 6]. Available from: http://methods.sagepub.com/book/multidimensional-scaling.

[51] Trochim W. Reliability of Concept Mapping. In 1993. Paper presented at the Annual Conference of the American Evaluation Association, Dallas, Texas, November 6, 1993. Available from: http://www.billtrochim.net/research/Reliable/reliable.htm.

[52] Nunnally J. Psychometric theory. 2d ed. New York: McGraw-Hill; 1978.

[53] Li M, Sum RKW. A meta-synthesis of elite athletes’ experiences in dual career development. Asia Pacific Journal of Sport and Social Science. 2017; 3;1–19. 10.1080/21640599.2017.1317481.

[54] Elliott S, Drummond MJN, Knight C. The experiences of being a talented youth athlete: Lessons for parents. Journal of Applied Sport Psychology. 2018;30(4):437–455. 10.1080/10413200.2017.1382019.

[55] Comeaux E, Harrison CK. A conceptual model of academic success for student–athletes. Educational Researcher. 2011;40(5):235–245. 10.3102/0013189X11415260.

[56] Harrison CK, Tranyowicz L, Bukstein S, McPherson-Botts G, Lawrence SM. I am what I am? The Baller Identity Measurement Scale (BIMS) with a Division I football team in American higher education. Sport Sci Health. 2014;10(1):53–58. 10.1007/s11332-014-0171-3.

[57] Lally PS, Kerr GA. The career planning, athletic identity, and student role identity of intercollegiate student athletes. Research Quarterly for Exercise and Sport. 2005;76(3):275–285. doi: 10.1080/02701367.2005.10599299 16270705

[58] Lupo C, Guidotti F, Goncalves CE, Moreira L, Doupona Topic M, Bellardini H, et al. Motivation towards Dual Career of European student-athletes. European Journal of Sport Science. 2015;15(2):151–160. doi: 10.1080/17461391.2014.940557 25145585

[59] Stambulova NB, Engström C, Franck A, Linnér L, Lindahl K. Searching for an optimal balance: Dual career experiences of Swedish adolescent athletes. Psychology of Sport and Exercise. 2015;21:4–14. 10.1016/j.psychsport.2014.08.009.

[60] Burlot F, Richard R, Joncheray H. The life of high-level athletes: The challenge of high performance against the time constraint. International Review for the Sociology of Sport. 2018 Mar;53(2):234–49. 10.1177/1012690216647196.

[61] Linnér L, Stambulova NB, Lindahl K, Wylleman P. Swedish university student-athletes’ dual career scenarios and competences. International Journal of Sport and Exercise Psychology. 2019 May 7;1–16. 10.1080/1612197X.2019.1611898.

[62] Thrower SN, Harwood CG, Spray CM. Educating and supporting tennis parents: an action research study. Qualitative Research in Sport, Exercise and Health. 2017 Oct 20;9(5):600–18. 10.1080/2159676X.2017.1341947.

[63] O’Neill M, Calder A, Allen B. Australian parents’ perceptions of the issues faced by their adolescent high performance sports children in balancing school and sport. Journal of Sports Pedagogy and Physical Education. 2015; 6(3):1–12. Available from: https://research.usc.edu.au/discovery/fulldisplay/alma99449141102621/61USC_INST:ResearchRepository.

[64] Coakley J. The Good Father: Parental Expectations and Youth Sports. Leisure Studies. 2006;25(2):153–63. 10.1080/02614360500467735.

[65] Aunola K, Sorkkila M, Viljaranta J, Tolvanen A, Ryba TV. The role of parental affection and psychological control in adolescent athletes’ symptoms of burnout. Journal of Adolescence. 2018;69. doi: 10.1016/j.adolescence.2018.10.001 30316020

[66] Krubbeltrang LS, Karen D, Nielsen JC, Olesen JS. Reproduction and opportunity: A study of dual career, aspirations and elite sports in Danish SportsClasses. International Review for the Sociology of Sport. 2020;55(1):38–59. 10.1177/1012690218789037.

[67] Sorkkila M, Aunola K, Ryba TV. A person-oriented approach to sport and school burnout in adolescent student-athletes: The role of individual and parental expectations. Psychology of Sport and Exercise. 2017;28:58–67. 10.1016/j.psychsport.2016.10.004.

[68] Sorkkila M, Ryba TV, Selänne H, Aunola K. Development of school and sport burnout in adolescent student‐athletes: A longitudinal mixed‐methods study. Journal of Research on Adolescence. 2020;30(S1):115–133. 10.1111/jora.12453. 30207416

[69] Pavlidis G, Gargalianos D. Structural support of high-performance athletes’ education supporting dual careers in Greece: Column editor:: Roger Jackson. Strategies. 2014;27(1):42–45. 10.1080/08924562.2014.863691.

[70] Pavlidis G, Gargalianos D. High performance athletes’ education: Value, challenges and opportunities. Journal of Physical Education & Sport. 2014;14:293–300. 10.7752/jpes.2014.02044.

[71] Haugen TA, Solberg PA, Foster C, Morán-Navarro R, Breitschädel F, Hopkins WG. Peak age and performance progression in world-class track-and-field athletes. International Journal of Sports Physiology and Performance. 2018;13(9):1122–1129. doi: 10.1123/ijspp.2017-0682 29543080

[72] Sal de Rellán‐Guerra A, Rey E, Kalén A, Lago‐Peñas C. Age‐related physical and technical match performance changes in elite soccer players. Scand J Med Sci Sports. 2019;29(9):1421–1427. doi: 10.1111/sms.13463 31099117

[73] Engh MH, Agergaard S, Maguire J. Established–outsider relations in youth football tournaments: An exploration of transnational power figurations between Scandinavian organizers and African teams. Soccer & Society. 2013;14(6):781–798. 10.1080/14660970.2013.843907.

[74] Barget E, Chavinier-Rela S. The analysis of amateur sports clubs funding: A European perspective. AJSPO. 2017;4(1):7–34. 10.30958/ajspo.4.1.1.

[75] Nichol AJ, Hall ET, Vickery W, Hayes PR. Examining the relationships between coaching practice and athlete “outcomes”: A systematic review and critical realist critique. International Sport Coaching Journal. 2019;6(1):13–29. 10.1123/iscj.2017-0105.

